# Overrepresentation of IL-17A and IL-22 Producing CD8 T Cells in Lesional Skin Suggests Their Involvement in the Pathogenesis of Psoriasis

**DOI:** 10.1371/journal.pone.0014108

**Published:** 2010-11-24

**Authors:** Pieter C. M. Res, Gamze Piskin, Onno J. de Boer, Chris M. van der Loos, Peter Teeling, Jan D. Bos, Marcel B. M. Teunissen

**Affiliations:** 1 Department of Dermatology, Academic Medical Center, University of Amsterdam, Amsterdam, The Netherlands; 2 Department of Pathology, Academic Medical Center, University of Amsterdam, Amsterdam, The Netherlands; New York University, United States of America

## Abstract

**Background:**

Although recent studies indicate a crucial role for IL-17A and IL-22 producing T cells in the pathogenesis of psoriasis, limited information is available on their frequency and heterogeneity and their distribution in skin *in situ*.

**Methodology/Principal Findings:**

By spectral imaging analysis of double-stained skin sections we demonstrated that IL-17 was mainly expressed by mast cells and neutrophils and IL-22 by macrophages and dendritic cells. Only an occasional IL-17^pos^, but no IL-22^pos^ T cell could be detected in psoriatic skin, whereas neither of these cytokines was expressed by T cells in normal skin. However, examination of *in vitro*-activated T cells by flow cytometry revealed that substantial percentages of skin-derived CD4 and CD8 T cells were able to produce IL-17A alone or together with IL-22 (i.e. Th17 and Tc17, respectively) or to produce IL-22 in absence of IL-17A and IFN-γ (i.e. Th22 and Tc22, respectively). Remarkably, a significant proportional rise in Tc17 and Tc22 cells, but not in Th17 and Th22 cells, was found in T cells isolated from psoriatic versus normal skin. Interestingly, we found IL-22 single-producers in many skin-derived IL-17A^pos^ CD4 and CD8 T cell clones, suggesting that *in vivo* IL-22 single-producers may arise from IL-17A^pos^ T cells as well.

**Conclusions/Significance:**

The increased presence of Tc17 and Tc22 cells in lesional psoriatic skin suggests that these types of CD8 T cells play a significant role in the pathogenesis of psoriasis. As part of the skin-derived IL-17A^pos^ CD4 and CD8 T clones developed into IL-22 single-producers, this demonstrates plasticity in their cytokine production profile and suggests a developmental relationship between Th17 and Th22 cells and between Tc17 and Tc22 cells.

## Introduction

Psoriasis is a chronic inflammatory skin disease of unknown etiology, characterized by T cell infiltrates and epidermal thickening, due to hyperproliferation of keratinocytes [1], [2], [3], [4]. For many years, psoriasis was considered to be a Th1-mediated disease, because of the relative increase of circulating and skin-residing IFN-γ-producing T cells [5], [6] and the activation of many IFN-γ-induced immune response genes [7]. However, since the discovery that IL-17A-producing CD4 T cells (Th17) are crucially involved in the pathogenesis of some mouse autoimmune diseases [8], [9], [10], and because psoriasis is often considered an autoimmune or autoinflammatory disorder, many investigators switched their attention to Th17 cells as possible main instigators of psoriasis. Th17 cells have as key features that they produce IL-17A and that IL-23 is important for their maintenance [11]. Several observations support the involvement of the IL-23/IL-17A pathway in the pathogenesis of psoriasis. Mice overexpressing IL23p19 develop severe inflammation of many organs, including the skin [12]. Intradermal injection of IL-23 in murine skin leads to a type of skin inflammation that more closely resembles the histopathological features of psoriatic skin than skin inflammation induced by IL-12, a key cytokine for Th1 development [13], [14]. Levels of mRNA for the IL-23p19 and common IL-12/IL-23p40 units, but not for the IL-12p35 unit, are increased in lesional skin of psoriasis patients [13], [15] and also at protein level IL-23 is more abundantly expressed [16]. Furthermore, sequence variation in the genes encoding the common IL-12/23p40 unit and IL-23R is associated with psoriasis [17], [18]. Finally, treatment with a neutralizing IL-12/23p40 antibody has proven to be a very effective therapeutic modality for psoriasis patients [19], [20], [21], [22].

With regard to IL-17A, we have previously demonstrated that many T cell clones from lesional psoriatic skin express IL-17A mRNA, that the IL-17A mRNA levels in psoriatic skin are much higher than in symptomless skin [23], and that IL-17A in combination with IFN-γ stimulates the production of inflammatory cytokines in keratinocytes [23]. IL-17A by itself induces the production of antibacterial peptides by keratinocytes, as well as angiogenesis, which is interesting to note as high levels of antibacterial peptides and hyperplasia of blood vessels are typical features of psoriatic skin [24], [25]. Also, clinical data support the involvement of Th17 cells in psoriasis, as early disease improvement in patients treated with the TNF-α inhibitor etanercept coincides in time with the reduction of Th17 gene products and downstream effector molecules [26].

IL-17F and IL-22 are other cytokines typically produced by Th17 cells and may also play a role in the induction of psoriasis. IL-17F has a strong homology with IL-17A and stimulates proinflammatory cytokine production by epithelial cells as well [27], whereas IL-22 has a keratinocyte proliferation-promoting capacity [25]. Intradermal injection of IL-23 in wild-type mice treated with IL-17A- or IL-22-blocking antibodies, or in IL-22 receptor-deficient mice, demonstrated that actually IL-22, but not IL-17A, is responsible for the induction of acanthosis [14]. IL-22 neutralizing antibodies also prevented the development of a psoriasis-like disease that is induced by the transfer of BALB/c CD4^pos^CD45RB^hi^ T cells into SCID mice [28]. Furthermore, IL-22 mRNA expression is upregulated in psoriatic skin lesions compared to normal skin [29] and recombinant IL-22 dose-dependently promotes acanthosis in reconstituted human epidermis *in vitro* [30], a feature most likely related to its ability to downregulate genes involved in keratinocyte differentiation [25]. Like IL-17A, IL-22 is able to upregulate the production of antimicrobial peptides by keratinocytes [31].

All these results point to a prominent role for the IL-23/IL-17A pathway in the etiology of psoriasis, and many investigators have speculated about a principal role for Th17 cells in particular. However, we and others have shown that, in addition to CD4 Th cells, IL-17A can also be produced by CD8 T cells, in this study referred to as Tc17 [23], [32]. CD8 T cells, which are activated in a MHC class I-restricted fashion, are overrepresented in the epidermis of lesional psoriatic skin [33], [34]. Coincidently, MHC class I HLA-Cw6 is one of the psoriasis susceptibility alleles [35], [36], suggesting that CD8 T cells may be involved in the pathogenesis of psoriasis. Nevertheless, the major focus of research has traditionally been on Th cells, thereby possibly underestimating the role of the CD8 subset. Information about the relative proportions of CD4 and CD8 T cells capable of IL-17A and IL-22 production present in psoriatic skin compared to normal skin is limited. To this end, we performed immunohistochemical double-stainings to determine the presence, nature, and distribution of IL-17 and IL-22 expressing cells in lesional psoriatic skin and healthy normal skin *in situ*. To analyze the double-stained sections we used the spectral imaging technique that offers the great advantage to accurately display the location and abundance of each individual chromogen, enabling to pinpoint co-localization. In addition, we isolated and stimulated T cells from psoriatic and normal skin and used 6 color flow cytometry to compare the percentages of IL-17A and IL-22 producing CD4 and CD8 T cells between both conditions. Finally, we cloned Th17 and Tc17 cells from psoriatic dermis and epidermis to study the heterogeneity and stability of their IL-17A and IL-22 cytokine production profile.

## Materials and Methods

### Ethics statement

The Medical Ethical Committee of the Academic Medical Center has approved the use of patient's material upon informed written consent for the enclosed study.

### Clinical material

Eight male patients (mean age 49; range 41–63) with active psoriasis vulgaris visiting the department of Dermatology at the Academic Medical Center in Amsterdam volunteered to participate in this study. None of the patients used any systemic therapy or phototherapy for at least 4 weeks prior to participation. The mean psoriasis area and severity index (PASI) of the patients was 9.0±4.5. All patients gave written informed consent before donating 5 mm punch biopsies from an active expanding plaque. Normal adult skin was obtained from healthy subjects undergoing plastic surgery of the breast or abdomen after informed consent.

### Double-staining procedure

Sequential double alkaline phosphatase (AP) staining was performed to identify IL-17^pos^ and IL-22^pos^ cells in 5 µm sections from formalin-fixed paraffin-embedded biopsies from lesional psoriatic or normal human skin. After dewaxing and heat-induced epitope retrieval with Tris-EDTA pH 9.0 (20 min, 98°C), specimens were stained first for IL-17 (3-step) or IL-22 (2-step) with a polymer detection procedure. Polyclonal goat IgG anti-human IL-17A, which has 10% cross-reactivity with IL-17F according to the manufacturer's data sheet, and monoclonal mouse anti-human IL-22 were purchased from R&D Systems Europe Ltd, Abingdon, UK. The incubation with anti-IL-17A/F (overnight, 4°C) was followed by incubations with rabbit anti-goat IgG and a PowerVision anti-rabbit IgG, AP-labeled polymer (ImmunoLogic, Duiven, Netherlands). The incubation with anti-IL-22 (overnight, 4°C) was followed by incubation with a PowerVision anti-mouse IgG, AP-labelled polymer (ImmunoLogic). Vector Blue (Vector Labs, Burlingame, CA, USA) was used as AP reaction product. Next, all immuno-reagents used in the first staining sequence were removed by a second heat-induced epitope retrieval step (10 min, 98°C), leaving the blue reaction product intact. Subsequently, the second AP staining was performed using Liquid Permanent Red (Dako, Glostrup, Denmark) as reaction product. To detect T cells and neutrophils we used rabbit anti-human CD3 (Thermoscientific/LabVision, Fremont, CA, USA) and rabbit anti-human myeloperoxidase (Dako), respectively, and PowerVision anti-rabbit IgG, AP-labeled polymer as second step. To detect dendritic cells and macrophages we used mouse mAb anti-human CD11c (Monosan, Uden, The Netherlands) and mouse mAb anti-human CD68 (Dako), respectively, and PowerVision anti-mouse IgG, AP-labeled polymer as second step. AP-labeled anti-tryptase (Chemicon/Bioconnect, Huizen, The Netherlands) was used to detect mast cells. Negative and ‘half’ double staining control experiments were performed with matched species or mouse isotype control reagents using similar immunoglobulin concentrations.

### Spectral imaging

The spectral imaging technique was applied for the exact determination of color-colocalization to analyze all blue and red double-stained specimens using the NUANCE™ camera system (Cambridge Research Instrumentation Inc., Woburn, MA, USA) (37). First, spectral libraries of single-red (Liquid Permanent Red) and single-blue (Vector Blue) were loaded, using “half” double staining control slides. Next, these libraries were applied to the double staining slides and subsequently the Liquid Permanent Red and Vector Blue reaction products could be spectrally unmixed in two individual images [37]. The Nuance software (version 2.4) allows for the creation of a fluorescent-like image with a pseudocoloring of the reaction product, as well as the exclusive imaging of co-localization.

### Isolation of skin T cells

Five mm punch biopsies were treated overnight at 4°C with 0.3% dispase II (Roche, Almere, Netherlands) in PBS to enable separation of the dermis and epidermis, which were subsequently cultured separately in culture medium (IMDM with 10% NHS) in 24-well plates to allow spontaneous migration of T cells from the skin tissue fragments. All wells were screened microscopically for the presence of T cells on a daily basis and a culture period of 6-7 days appeared to be optimal to allow maximum numbers of T cells to migrate from the skin fragments.

### FACS analysis

The following antibodies were used: AF-647-conjugated anti-human IL-17A was obtained from eBioscience (San Diego, CA, USA) and PE-conjugated anti-human IL-22 from R&D Systems (Abingdon, UK). From BD Biosciences (Mountain View, CA, USA) we purchased FITC-conjugated anti-human IFN-γ, PerCP-conjugated anti-CD3, PE-Cy7-conjugated anti-CD4, and APC-Cy7-conjugated anti-CD8. Skin derived T cells were stimulated with 100 ng/ml PMA and 1 µg/ml ionomycin in IMDM supplemented with 10% normal human type AB serum in a 24 well plate for 5 hrs, the last 4 hr in the presence of Golgiplug (BD Biosciences). A six color FACS analysis was performed as follows. Cell surface staining with antibodies against CD3, CD4 and CD8 added in FACS buffer (PBS with 1% BSA and 0.05% sodium azide) at predetermined optimal concentrations was performed before fixation of cells for 15 min with 4% paraformaldehyde. Cells were washed with FACS buffer twice and one more time with PERM/WASH medium (BD Biosciences) to permeabilize cells to allow intracellular staining. Cells were subsequently incubated with antibodies against the cytokines and again with antibodies to CD3, CD4 and CD8. For this purpose, appropriate dilutions of these antibodies in PERM/WASH were incubated with the cells for 30 min, next cells were washed twice with PERM/WASH and one more time in FACS buffer and after resuspension in FACS buffer measured with a FACS Calibur (BD Biosciences). Data analysis was performed on cells within an electronic gate set to contain the lymphocytes on basis of their forward versus sideward scatter pattern. As a negative control, populations were left unstimulated and subsequently stained using the same protocol as described above.

### Cloning of dermal Th17 and epidermal CD8 T cells

IL-17A-producing T cells were isolated as described by Streeck et al [38] with some minor modifications. The IL-17A capture complex was freshly made every time just prior to use as follows: 2 µL of a biotin-labeled CD45 antibody (clone HI30; Caltag/Invitrogen, CA) were combined in an eppendorf tube with 20 µL (0.5 mg/mL) of biotin-labeled IL-17A antibody (Ebiosciences) and mixed thoroughly. Next, 2 µL (5 mg/mL) of a free avidin solution (Invitrogen, Paisley, UK) were added and immediately mixed well. The complex was incubated for 10 min at room temperature and was mixed well again before use. Dermal T cells stimulated for 3.5 h with PMA and ionomycin, were washed with ice cold PBS and resuspended in 100 µL of PBS plus 2% human serum, whereafter the 24 µL of IL-17A capture complex were added. After labeling for 15 min on ice, the volume was adjusted to 20 ml with IMDM plus 10% human serum and cells were cultured in a P75 culture flask (Corning Life Sciences, Amsterdam, The Netherlands) for an additional two hours. Every 15 min the flask was gently shaken. Next, cells were collected and double stained with a fluorochrome-conjugated IL-17A antibody (Ebiosciences; binding a different IL-17A epitope than the capture antibody) to label the captured IL-17A, and with anti-CD4 and CD8 fluorochrome conjugated antibodies. The IL-17A^pos^ CD4 T cells present within the characteristic lymphocyte gate of the forward versus sideward scatter plot were subsequently sorted by FACS Aria equipment (BD Biosciences) and cloned at a concentration of 1 cell/well in a 96-wells round-bottom plate. The sorted cells were cultured in a volume of 200 µL in IMDM plus 10% human serum, PHA (500 ng/ml), recombinant IL-2 (30 U/ml; Novartis) and IL-15 (10 ng/ml; Pelikine) in the presence of a feeder mixture consisting of 5×10^4^ allogeneic PBMC of two unrelated donors and 5×10^3^ EBV-transformed B cells, irradiated with 4 Gy and 8 Gy, respectively. Due to very limited numbers the IL-17A release-capture method could not be used for the cloning of T cells derived from epidermal lesional skin. Unstimulated epidermal T cells were cloned on basis of CD8 expression, without prior assessment of their IL-17A expression potential. Wells were screened microscopically for the presence of T cell outgrowth. Cells from positive wells were subsequently expanded in larger wells using the same stimulatory culture conditions but with more feeder cells. After return to a resting phase, cells were analysed for cytokine expression, as described above.

### Statistical analysis

Statistical analysis was done using the Student t test, using SPSS software, taking p<0.05 as significant.

## Results

### Abundant IL-17 and IL-22 expression by non-T cells in lesional psoriatic skin

**Figure 1:**
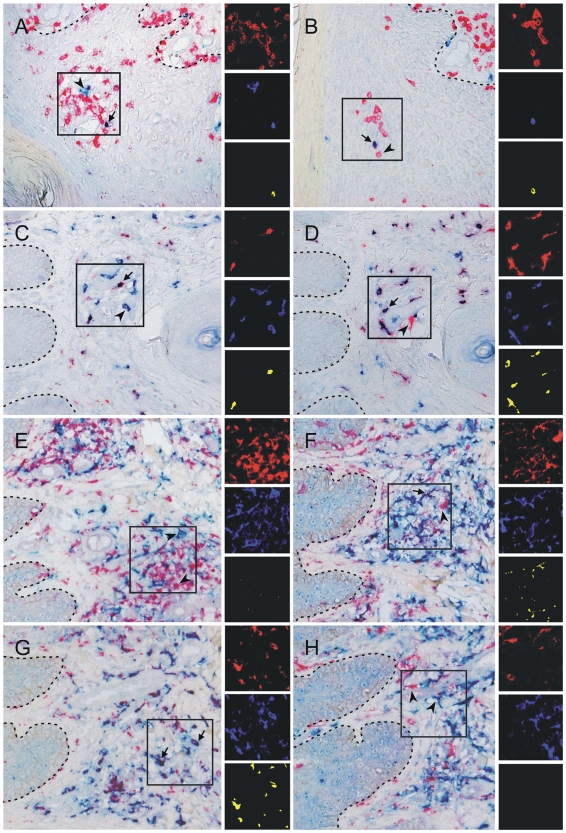
IL-17 and IL-22 expression in psoriatic skin. Microscopic detail of lesional psoriatic skin stained for IL-17 (A–D) or IL-22 (E–H) in combination with CD3 (A, B, E), myeloperoxidase (C), tryptase (D, H), CD11c (F), or CD68 (G). Cytokines are visualized in blue and all different cell markers in red. Each set of three small pictures on the right side represents a composite fluorescent-like image in pseudo-colors of the rectangle-marked area and was obtained after unmixing the individual colors red and blue with spectral imaging. Colocalization of red and blue is indicated in yellow. Spectral imaging analysis of this double-stained series of sections clearly shows the presence of an occasional IL-17^pos^ T cell in psoriatic lesions, whereas IL-22^pos^ T cells were absent. The IL-17 expression in psoriatic skin was predominantly confined to neutrophils and mast cells and IL-22 was highly expressed in many dendritic cells and in most macrophages. The figure is representative of the results from three different donors. Examples of double-stained cells are indicated with an arrow and single-stained cells with an arrowhead. Original magnification 200×.

In order to determine the presence and distribution of IL-17 producing T cells *in situ*, we performed double staining on skin sections from lesional psoriatic skin and healthy normal skin. Spectral digital imaging was used for objective determination of colocalization. Whereas clear IL-17^pos^ and CD3^pos^ cells could be observed in lesional psoriatic skin (Figures 1A and 1B), only an occasional T cell appeared to co-express IL-17. Nevertheless, the presence of IL-17^pos^ T cells seemed to be a specific feature of lesional skin, as in sections from normal skin no double stained cells could be detected (data not shown). In order to identify the IL-17^pos^ non-T cells, we performed additional double stainings. We found that the majority of neutrophils (myeloperoxidase positive) and virtually all mast cells (tryptase positive) in psoriatic skin were IL-17^pos^ (Figures 1C and 1D). We could also detect IL-17^pos^ cells in normal skin, which all appeared to be mast cells and not neutrophils (data not shown).

Because IL-22 is generally considered as another typical Th17-related cytokine, we determined the presence and distribution of IL-22 as well. Although we found a clear expression of IL-22 in a large number of cells in the inflammatory infiltrate in psoriatic skin, we could not detect any co-expression with CD3 (Figure 1E). Additional double stainings revealed that IL-22 was present in some dendritic cells (CD11c) and in the majority of macrophages (CD68) (Figures 1F and 1G, respectively), but neither in mast cells (Figure 1H) nor in neutrophils (not shown). IL-22 expression was also present in the same populations in normal skin (data not shown). T cells producing IL-17 and IL-22 have been suggested to play a prominent role in the pathogenesis of psoriasis and indeed we were able to detect these cytokines in the psoriatic lesions *in situ*, however, the remarkable observation of our immunohistochemical analysis is that the vast majority of the cells that express these cytokines are non-T cells.

### Proportional increase of Tc17 but not Th17 cells in psoriatic skin

**Figure 2:**
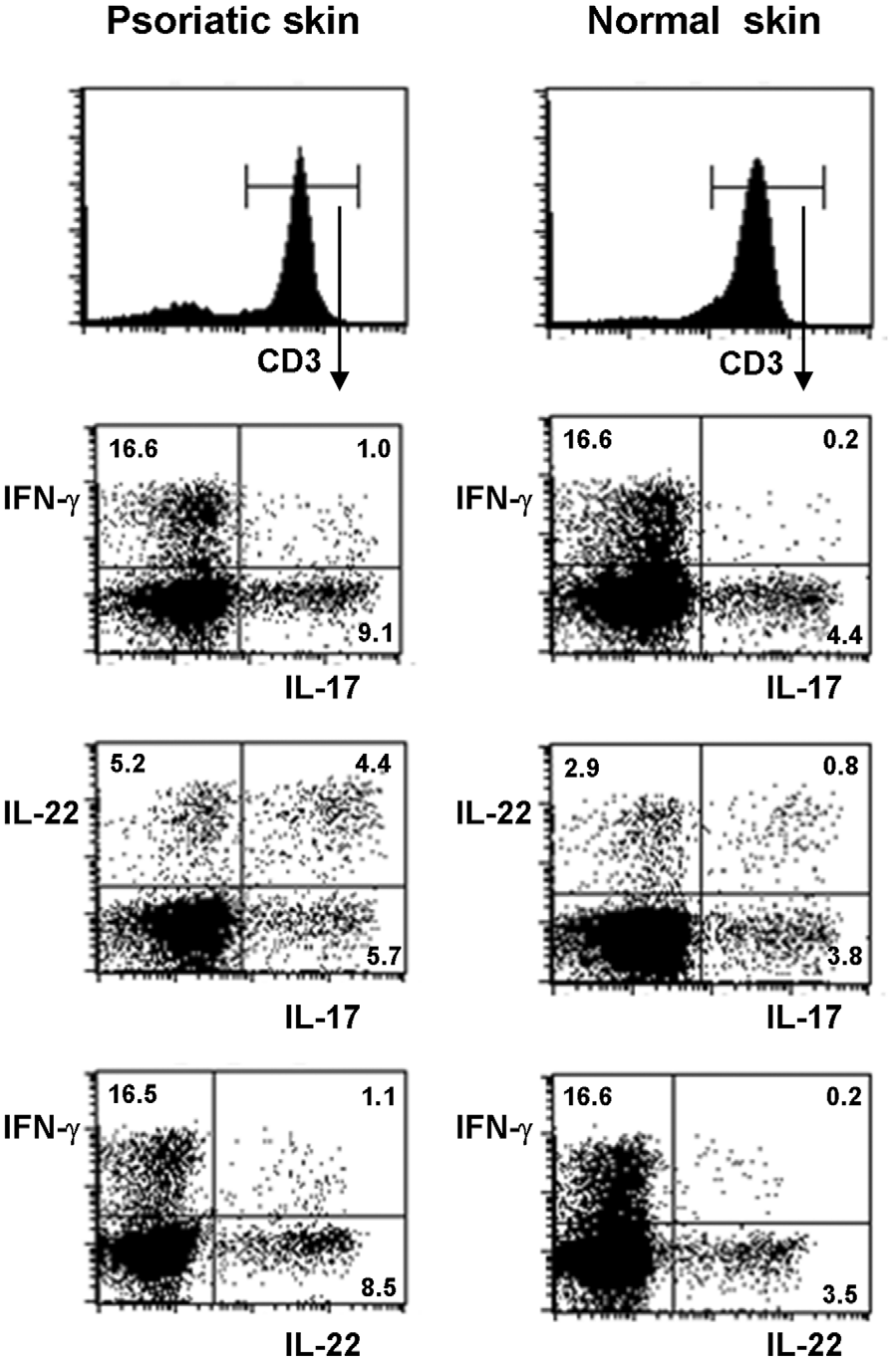
Cytokine expression in skin derived dermal T cells after in vitro stimulation. T cells with the ability to produce IL-17A and/or IL-22 and/or IFN-γ are present in the dermis of both psoriatic and normal skin. Bulk T cells derived from the dermis were stimulated with PMA and ionomycin and stained for cell surface expression of CD3, CD4 and CD8 and intracellular expression of IL-17A, IL-22 and IFN-γ. Dot-plots contain the CD3 cells within the lymphocyte gate. This analysis shows the presence among dermal CD3 T cells of both normal and psoriatic skin of single and double producers of IL-17A, IL-22 and IFN-γ. Results are representative for eight independent experiments.

**Figure 3:**
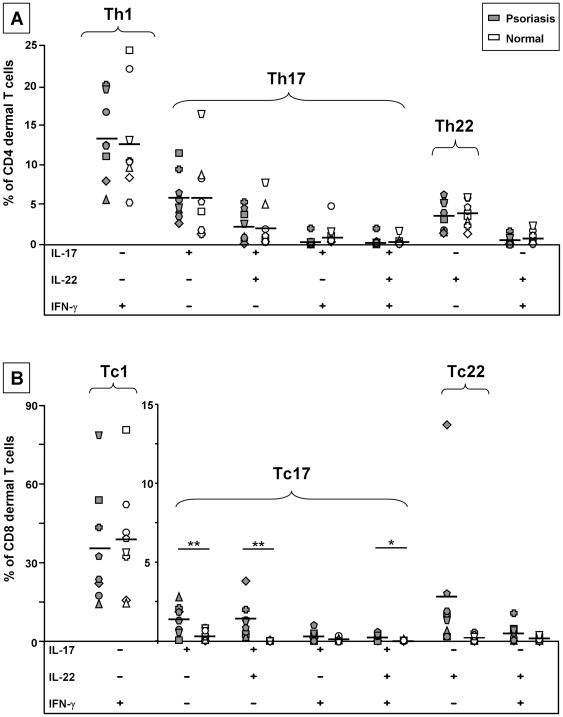
Cytokine profiles of dermis-derived CD4 and CD8 bulk T cell populations. Relative contribution of T cells with different expression profiles of IL-17A, IL-22 and IFN-γ production among *in vitro* activated dermal CD4 T cells (a) and CD8 T (b) from psoriatic skin (closed symbols) versus normal skin (open symbols). Each series of a particular symbol represents data from one individual. Specifically T cells with the ability to produce IL-17A and/or IL-22 are present in increased percentages of the CD8 T cell population in lesional skin compared to normal skin. *P<0.05; **P<0.01 Depicted data show the results obtained by six color flow cytometry of the eight independent experiments.

We were able to detect only an occasional IL-17^pos^ and no IL-22^pos^ T cell in lesional psoriatic skin *in situ*, but one should consider that T cells only temporarily express cytokines upon activation, that intralesional T cells are unlikely to be all in the same phase of activation, and that T cells are most likely in a resting stage in normal skin. Consequently, *in situ* analysis of cytokine expression by T cells probably represents an underestimation of the percentage T cells with the ability to produce a cytokine of interest. As an alternative approach to gain insight into the percentages of IL-17A and IL-22 producing T cells, we isolated T cells which spontaneously migrated from cultured dermal and epidermal skin fragments and determined their ability to produce these cytokines upon mitogenic activation *in vitro*. In addition, we also studied the production of IFN-γ. Six color flow cytometry was performed to study the simultaneous expression of CD3, CD4, CD8, IL-17A, IL-22 and IFN-γ by individual cells. Representative cytokine expression profiles of CD3 populations from the dermal component of psoriatic and normal skin are shown in the FACS dot plots in Figure 2. The proportions of dermal CD4 and CD8 T cells expressing distinct cytokine profiles are shown in Figure 3. Dermal T cells derived from psoriatic skin, as well as from normal skin, consistently contained a substantial percentage of IL-17A producers, indicating that the presence of IL-17A^pos^ T cells is not restricted to lesional skin (Figures 2 and 3). Some IL-17A^pos^ T cells coproduced IL-22, consistent with what has been described for mouse as well as human Th17 cells. On the other hand, all dermal T cell samples from both lesional and normal skin contained T cells which produced IL-22 in the absence of IL-17A and IFN-γ, representing the recently defined Th22 cells and their CD8 “Tc22” counterparts (Figures 2 and 3). Only a minority of IL-17A and/or IL-22 producing T cells displayed IFN-γ production as well and could be distinguished in most samples (Figures 2 and 3). T cells producing IFN-γ in the absence of IL-17A and IL-22, in our study referred to as Th1 and Tc1 cells, accounted for the highest percentage of dermal T cells in both lesional and normal skin (Figures 2 and 3).

**Figure 4:**
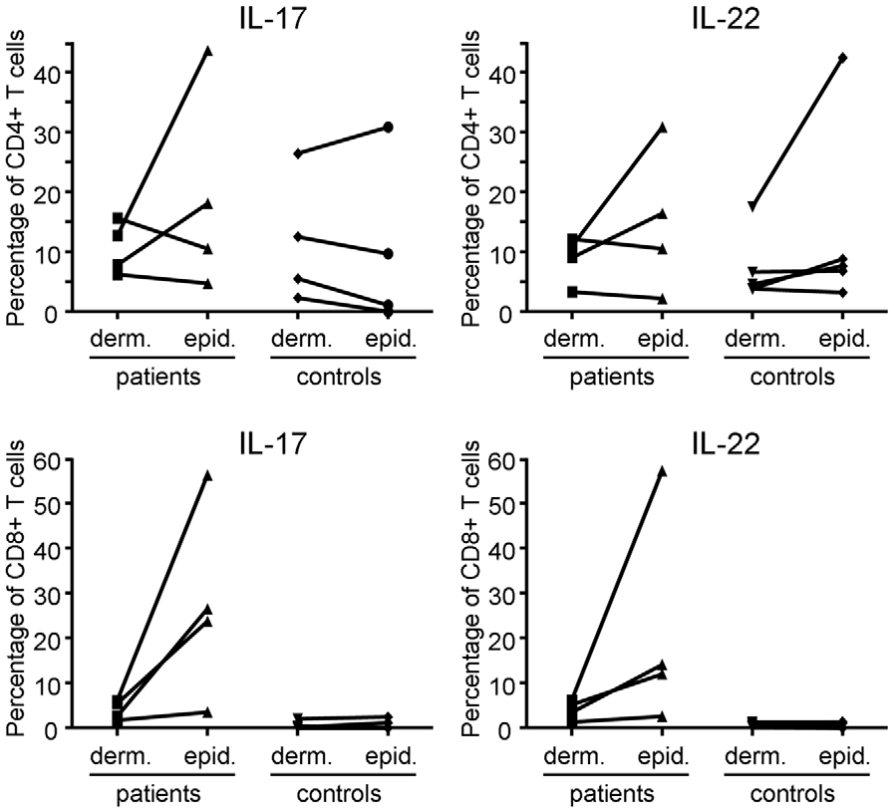
Proportions of IL-17A and IL-22 producing T cells in the epidermis versus dermis. The increase in the percentages of Tc17 and IL-22 producing CD8 T cells is even more pronounced in the psoriatic epidermis than in the psoriatic dermis, whereas normal epidermis and dermis are almost devoid of such T cells (n = 4).

When the cytokine profiles of dermal CD4 and CD8 T cell subsets were analyzed separately, we found, surprisingly, that the proportions of Th17 cells were very similar in psoriatic dermis compared to normal dermis (Figure 3A). This held true for Th17 cells that only produced IL-17A (psoriatic skin: 5.94%±1.13; normal skin: 5.87%±1.83%) and for Th17 cells that coproduced IL-22 (psoriatic skin: 2.36%±0.72%; normal skin: 2.05%±0.95%) or IFN-γ (psoriatic skin: 0.33%±0.12%; normal skin: 0.84%±0.41%). Obviously, in contrast to the percentage, the absolute number of Th17 cells is still much higher in psoriatic skin as it is more densely populated with T cells [6]. In both psoriasis patients and healthy individuals the total percentage of Th17 cells exceeded that of Tc17 cells (psoriatic skin: Th17 8.89%±1.88%, Tc17 3.39%±0.70%; normal skin: Th17 9.18%±3.13%; Tc17 0.35%±0.17%), which is consistent with our data on IL-17A expression by T cells in peripheral blood (unpublished observation). Remarkably, the dermal CD8 T cell population from psoriatic skin contained a significantly increased percentage of Tc17 cells (psoriatic skin: 3.39%±0.70%; normal skin: 0.35%±0.17%). This was not only the case for the IL-17A single producers, but also for Tc17 cells coproducing IL-22 (Figure 3B). Moreover, and further underlining a possible role of Tc17 cells in the pathogenesis of psoriasis, the epidermis contained an even 2 to 10 fold higher frequency of Tc17 cells than the adjacent dermis. This was observed in all four psoriasis patients from whom we had obtained enough epidermal T cells for analysis (Figure 4). In contrast, the population of T cells derived from the epidermis and adjacent dermis of normal skin contained similar low levels of Tc17 cells. Th17 cells were present in substantial numbers in the epidermis from both psoriatic and normal skin, but no consistent pattern of increase/decrease relative to the corresponding dermis was observed (Figure 4).

### Proportional increase of IL-17A^neg^ IFN-γ ^neg^ IL-22^pos^ CD8 T cells in psoriatic skin

Because of its property to promote acanthosis, IL-22 is highly relevant for the pathogenesis psoriasis. Although IL-22 has been associated with Th17 cells, recent publications indicate that IL-22 producing CD4 T cells exist that lack concomitant expression of IL-17A and IFN-γ and may represent a distinct functional T cell subset, named Th22 [39], [40]. In analogy, IL-22 producing CD8 T cells that lack IL-17A and IFN-γ can be termed Tc22. The dermal T cells of lesional psoriatic and normal skin contained similar percentages of Th22 cells (psoriatic skin: 3.70%±0.68%; normal skin: 3.90%±0.60%), but remarkably, the frequency of Tc22 cells was substantially increased in lesional skin (psoriatic skin: 2.85%±1.58%; normal skin 0.16%±0.07%). One patient was remarkable in this respect as 14% of the skin derived dermal CD8 T cells consisted of Tc22 cells, suggesting that a specific expansion of these T cells has taken place in the inflamed skin of this patient. As was the case with IL-17A^pos^ CD8 T cells, also the percentage of IL-22^pos^ CD8 T cells was much higher in the epidermis than in the adjacent dermis of psoriatic skin, but not in normal skin (Figure 4), underlining a possible role of IL-17A and IL-22 producing CD8 T cells in the pathogenesis of psoriasis. With regard to the well-known prominent role of IFN-γ in the etiology of this disease, we unexpectedly observed no increased frequencies of IFN-γ producing CD4 or CD8 T cells in the dermis of psoriasis patients compared to healthy skin donors. A comparison of IFN-γ expression in epidermal versus adjacent dermal T cell populations was only determined for three patients. In two of them the percentages of IFN-γ^pos^ T cells in both CD4 and CD8 subsets were similar, whereas in one patient there was about a two fold increase in the percentage of both CD4 and CD8 IFN-γ expressing T cells in the epidermal compared to the dermal compartment (data not shown).

### Cells with a Th22 and Tc22 cytokine profile derived from Th17 and Tc17 cells, respectively

**Figure 5:**
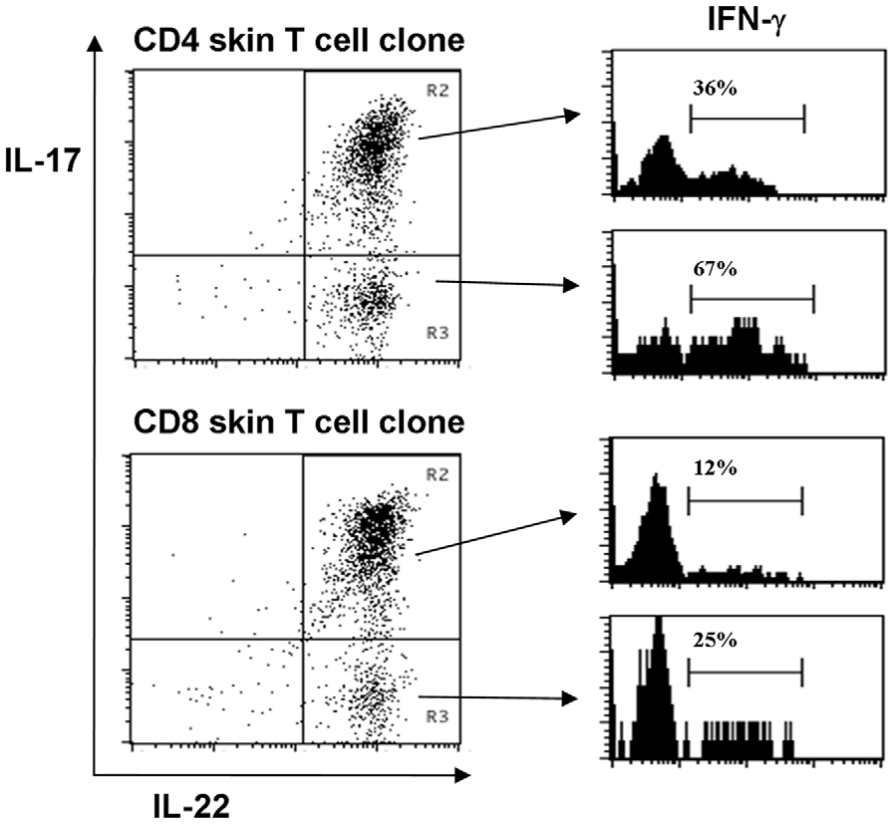
Cytokine profile of psoriatic skin derived Th17 and Tc17 clones. IL-17A^pos^ CD4 and CD8 T cell clones (Th17 and Tc17, respectively) from psoriatic skin have the ability to give rise to a proportion of IL-22 producing cells that lack IL-17A and IFN-γ expression (the putative Th22 and Tc22, respectively). IL-17A^pos^ CD4 dermal T cells and CD8 epidermal T cells derived from psoriatic skin were cloned and subsequently assayed for intracellular IL-17A, IL-22, and IFN-γ after PMA ionomycin stimulation. Representative examples of CD4 and CD8 T cell clones are given. Part of the cells within the CD4 Th17 clone expressed IL-22, but lacked both IL-17A (right-bottom quadrant in the upper dot-plot) and IFN-γ expression (associated histogram indicated by arrow), a cytokine pattern typical of Th22 cells. Likewise, part of the cells of the CD8 Tc17 clone (bottom dot-plot) lacked IL-17A and IFN-γ expression, which is a Tc22 cytokine profile.

One may question whether the cytokine patterns of the skin-infiltrating T cells are stable and transferred to the progeny after cell division. To investigate this for IL-17A producers in particular, we cloned psoriatic dermal Th17 cells which were purified by FACS sorting on the basis of an IL-17A release-capture assay. This technique could not be used when we cloned T cells from the corresponding epidermis, because of the limited number of epidermal T cells. In that case we purified T cells on basis of CD8 cell surface expression and twelve out of fifteen of the epidermal CD8 T cell clones turned out be IL-17A producers, consistent with our observation that epidermal CD8 T cells from psoriatic skin contain high percentages of Tc17 cells. The remaining three epidermal T cell clones expressed IFN-γ only, without IL-17A or IL-22, thus representing Tc1 clones. All cells within individual clones displayed the same T cell receptor V-beta family usage confirming clonality (data not shown). Twelve CD4 T cell clones, each originating from a different single IL-17A-expressing T cell, were found to still produce IL-17A after culture for one month. Unexpectedly however, in five of these Th17 clones a variable proportion (range 5–25%) of cells did produce IL-22 but lacked IL-17A, and in addition, part of these IL-17A^neg^IL-22^pos^ T cells lacked IFN-γ as well, indicating that some cells had acquired a Th22 profile (Figure 5). This suggests a developmental relationship between Th17 and Th22 cells and it is not inconceivable that Th22 cells may originate from Th17 precursors *in vivo*. Likewise, a proportion of cells with a Tc22 cytokine profile were found in six out of twelve of the IL-17A producing epidermal CD8 T cell clones derived from the same skin biopsy (Figure 5). Taken all together, our results suggest that CD4 and CD8 T cells in psoriatic skin have a certain degree of plasticity in their cytokine production pattern.

## Discussion

Because recent experimental findings suggest an important role for IL-17A and IL-22 producing T cells in the pathogenesis of psoriasis, we focused in this study on these types of T cells and studied their relative proportion in the CD4 and CD8 skin resident T cell population in psoriatic skin and normal skin. Thus far, most studies have speculated about a possible role for Th17 cells, i.e. CD4 T cells which, in addition to key cytokine IL-17A, also have been described to be able to produce IL-22. In contrast to expectations, we clearly showed that the dermis of psoriatic and normal skin contain similar frequencies of Th17 cells. This observation does not necessarily imply, however, that these cells have no role in the pathogenesis of psoriasis, but only suggest that Th17 cells do not expand in the lesional skin. On the other hand, because the density of T cells is much higher in psoriasis lesions [6], our results also imply that psoriatic skin does contain an increase in absolute numbers of Th17 cells per mm^2^. Of note, we found that percentages of Th17 cells are generally higher in the skin than in peripheral blood in both patients and healthy individuals (unpublished observation), which would indicate that Th17 cells preferentially home into the skin.

To our surprise, our results show unequivocally that, compared to normal skin, psoriatic skin contains a T cell infiltrate with a significantly increased percentage of Tc17 cells, i.e. CD8 T cells with the ability to produce IL-17A alone or in combination with IL-22. This indicates that this subset either shows a preferential homing to the lesions in psoriasis patients or proliferates at these sites after being activated, possibly as a result of recognition of locally presented antigen(s). This selective increase in the proportion of Tc17 cells in psoriatic skin, which is even more striking in the epidermis than the dermis, reinforces the concept that CD8 T cells may be important in the pathogenesis of psoriasis, as has been suggested earlier [33], [34]. Psoriatic epidermis is known to contain a significantly higher CD8 to CD4 T cell ratio than the dermis [34], [41] and also a strong association of psoriasis with HLA-Cw6 has been reported [35], [36], a MHC class I molecule involved in antigen presentation to CD8 T cells. In this respect, it is interesting to note that our patient with the relatively high proportion of IL-17A and/or IL-22-producing CD8 dermal T cells coincidently has a HLA-Cw6 phenotype (data not shown). It is tempting to speculate that these CD8 T cells may have responded specifically to (auto)-antigenic peptides presented on skin cells in the context of HLA-Cw6. With regard to this concept, it is interesting to note that CD8 skin homing, peripheral blood T cells in HLA-Cw6^pos^ psoriasis patients were shown to display an increased reactivity (IFN-γ production) against peptides derived from keratin 17, which is produced by keratinocytes at elevated levels in psoriatic skin [42]. These peptides were chosen on basis of homology between keratin 17 and streptococcal M protein and selected for HLA-Cw6 binding. Since psoriasis is sometimes initiated or exacerbated by a throat infection with streptococci and M protein is an important antigenic determinant, the authors speculate that T cells triggered by streptococcal proteins can cross-react with components from the lesional skin [42]. It would be interesting to find out whether psoriatic plaques derived CD8 T cells also show keratin 17 directed responses.

Recently, also other studies have compared IL-17A producing T cells derived from psoriatic and normal skin. One group reported a similar percentage of IL-17A^pos^ CD3 T cells in psoriatic dermis (mean 6% versus 7% in our study), but they found a much lower percentage of IL-17A^pos^ CD3 T cells (0.5% versus 6%) and IL-17A^pos^ CD4 T cells (1% versus 9%) in normal dermis [25], [43]. In another study, data were expressed as absolute numbers of Th17 or Tc17 cells per mm^2^ skin, which were both increased in psoriatic dermis [44]. However, it was left unmentioned whether also in that study only the Tc17 cells displayed a proportional increase in the psoriatic dermal T cell population. Interestingly, and also in line with our results, it was reported that the increase of Tc17 cells in absolute cell numbers was most pronounced in the psoriatic epidermis, and that Tc17 cells are virtually absent in normal epidermis and dermis [44]. In contrast to their findings, we observed that the epidermis contains substantial numbers of Th17 cells in both patients and healthy controls.

We found that part of CD4 as well as CD8 T cells from lesional psoriatic skin and normal skin produced IL-22 in the absence of IL-17A. The vast majority of IL-17A^neg^IL-22^pos^ cells turned out to be IFN-γ ^neg^ as well. Two recent publications have shown that CD4 T cells with such features represent a functionally distinct Th22 subset that is different from Th17 cells [39], [40]. Whether CD8 T cells that generate IL-22 without IL-17A and IFN-γ also make up a separate Tc22 cell subset still awaits further investigation. Nevertheless, our finding that the frequency of Tc22 cells was also increased in psoriatic dermis may well be of importance in the pathogenesis of psoriasis, because of the keratinocyte proliferation-promoting activity of this cytokine. The origin of IL-22 single-producing T cells is unknown to date, but our results suggest that at least part of these cells may derive from Th17 and Tc17 cells, as we found that some of our IL-17A producing CD4 and CD8 T cell clones contained a proportion of cells producing IL-22 but lacking IL-17A and IFN-γ. Apparently, at least *in vitro*, daughter cells from IL-17A producing T cells can lose the capacity to express IL-17A and develop into IL-22 single-producing T cells. In this respect, it is interesting to note that the patient with the highest percentage of Tc22 cells also contained the highest percentage of Tc17 cells in the bulk dermal T cell population.

Many studies have addressed the potential role of IFN-γ in the etiology of psoriasis. Elevated IFN-γ mRNA levels in psoriatic skin are indicative for a local production of this cytokine and can explain the observed upregulation in the expression of many immune response genes in psoriatic lesions. In addition, IFN-γ can stimulate keratinocytes and antigen-presenting cells to produce IL-1β and IL-23 and thus promote the expansion of IL-17A producing T cells. Although lesional psoriatic skin does contain larger numbers of cutaneous T cells than normal skin ― and accordingly more IFN-γ producing T cells ― we did not find a proportional increase in the percentage of IFN-γ expressing T cells in psoriatic dermis compared to normal dermis, irrespective of co-expression of IL-17A and/or IL-22. Comparison of the T cells from the psoriatic epidermis versus the adjacent dermis revealed an increase in the percentages of IFN-γ^pos^ T cells in both CD4 and CD8 subsets in the psoriatic epidermis in one out of three patients. Larger numbers of experiments are necessary to evaluate the contribution of epidermal IFN-γ producing T cells in the development of psoriasis.

In our attempt to identify IL-17^pos^ T cells in skin *in situ*, we demonstrated that numerous IL-17^pos^ cells were present in psoriasis lesions, but that only an occasional IL-17^pos^ cell could be identified as a T cell, suggesting that at the time of biopsy taking only a minor part of the IL-17^pos^ T cells present in the psoriatic skin were activated. We believe that we did not stain receptor-bound IL-17, as keratinocytes, which do express IL-17 receptors, were not stained. The abundant expression of IL-17 we found by immunohistochemistry is consistent with the high levels of IL-17 mRNA detected previously by RT-PCR [23], [24]. Our FACS data showed that, even after optimal stimulation *in vitro*, IL-17A expressing T cells generally represent less than 10% of the dermal T cell population from psoriatic skin. In addition, it is not to be expected that all Th17 and Tc17 cells will be simultaneously and/or continuously activated in skin *in situ*, therefore the presence of low numbers of IL-17^pos^ T cells in the skin sections appears reasonable. It was quite remarkable to observe that mast cells and neutrophils were the major cell types stained with our polyclonal anti-IL-17 antibody in the inflamed skin. This same reagent was used in recent studies to demonstrate that in the inflamed synovium of rheumatoid arthritis patients and in lesional tissue of atherosclerotic patients mast cells also represent an important source of IL-17 expression [45], [46]. Mast cells have been reported to produce IL-17F but not IL-17A and since our polyclonal Ab that recognizes IL-17A has crossreactivity to IL-17F according to the manufacturer, it is likely that a considerable part of the IL-17 we detected in human skin mast cells was IL-17F actually [46], [47]. On the same line, it has been reported that IL-17A producing T cells most often also produce IL-17F, sometimes even as a heterodimer, and also neutrophils can produce both IL-17A and IL-17F [48]. As concerned the IL-17^pos^ T cells and neutrophils in the stained skin sections, we do not know whether IL-17A or IL-17F or both were detected. Both cytokines are proinflammatory and may have a role in the pathogenesis of psoriasis, as both have features in common among others their ability to activate keratinocytes [49], [50].

Another surprising observation from our double-stained skin sections was that IL-22 was solely detected in non-T cells belonging to the myeloid lineage such as CD68^pos^ macrophages and CD11c^pos^ dendritic cells. Psoriatic lesions are not only characterized by an increase in T cells, but also in CD11c^pos^ dendritic cells. This increase specifically concerns the CD1c^neg^ TNF-α and iNOS expressing so-called “inflammatory” or TipDC and not the CD1c^pos^ dermal DC, which are also present in normal skin [51]. At the moment we do not know whether our CD11c^pos^IL-22^pos^ cells are TipDC or dermal DC.

As both IL-17A and IL-22 have been implicated as crucial cytokines in the pathogenesis of psoriasis, our immunohistochemistry data suggest that, in addition to T cells, also other IL-17^pos^ and/or IL-22^pos^ cells may be relevant to this chronic skin disease and possible targets for intervention in psoriasis therapy. As mentioned earlier, the increase we have observed in the percentage of CD8 T cells with the potential to produce IL-17A and/or IL-22 in psoriatic skin suggests an antigen-driven expansion of these T cells at the site of inflammation, but their precise role in the pathogenesis of psoriasis still requires more investigation. It could well be that activated Tc17 cells and Tc22 cells, in addition to IL-17A and IL-22, produce other yet undiscovered mediators that are involved in the inflammatory process in the psoriasis plaques. Recently, the potential role of CD8 T cells in the etiology of other human diseases has been reevaluated, such as in systemic sclerosis [52], multiple sclerosis [53] and atopic dermatitis [32]. Here we provide support for involvement of Tc17 and Tc22 cells in the pathogenesis of psoriasis underlining that the common interest in these cells has wrongly been largely ignored. Exploring their antigen reactivity may well be of great importance to find out whether these CD8 T cell subsets respond to components from the inflamed skin in a HLA-Cw6 restricted fashion, which would make these cells even more relevant as potential targets for immunotherapy.

## References

[1] Teunissen MB, Piskin G, Res PC, de Groot M, Picavet DI (2007). State of the art in the immunopathogenesis of psoriasis.. G Ital Dermatol Venereol.

[2] Bos JD, de Rie MA, Teunissen MB, Piskin G (2005). Psoriasis: dysregulation of innate immunity.. Br J Dermatol.

[3] Bos JD (2007). Psoriasis, innate immunity, and gene pools.. J Am Acad Dermatol.

[4] Nickoloff BJ, Xin H, Nestle FO, Qin JZ (2007). The cytokine and chemokine network in psoriasis.. Clin Dermatol.

[5] Austin LM, Ozawa M, Kikuchi T, Walters IB, Krueger JG (1999). The majority of epidermal T cells in Psoriasis vulgaris lesions can produce type 1 cytokines, interferon-gamma, interleukin-2, and tumor necrosis factor-alpha, defining TC1 (cytotoxic T lymphocyte) and TH1 effector populations: a type 1 differentiation bias is also measured in circulating blood T cells in psoriatic patients.. J Invest Dermatol.

[6] Szabo SK, Hammerberg C, Yoshida Y, Bata-Csorgo Z, Cooper KD (1998). Identification and quantitation of interferon-gamma producing T cells in psoriatic lesions: Localization to both CD4(+) and CD8(+) subsets.. J Invest Dermatol.

[7] Zhou XH, Krueger JG, Kao MCJ, Lee E, Du FH (2003). Novel mechanisms of T-cell and dendritic cell activation revealed by profiling of psoriasis on the 63,100-element oligonucleotide array.. Physiol Genomics.

[8] Cua DJ, Sherlock J, Chen Y, Murphy CA, Joyce B (2003). Interleukin-23 rather than interleukin-12 is the critical cytokine for autoimmune inflammation of the brain.. Nature.

[9] Langrish CL, ChenY, Blumenschein WM, Mattson J, Basham B (2005). IL-23 drives a pathogenic T cell population that induces autoimmune inflammation.. J Exp Med.

[10] Murphy CA, Langrish CL, ChenY, Blumenschein C, McClanahan T (2003). Divergent pro- and Antiinflammatory roles for IL-23 and IL-12 in joint autoimmune inflammation.. J Exp Med.

[11] Stritesky GL, Yeh N, Kaplan MH (2008). IL-23 Promotes Maintenance but Not Commitment to the Th17 Lineage.. J Immunol.

[12] Wiekowski MT, Leach MW, Evans EW, Sullivan L, Chen SC (2001). Ubiquitous transgenic expression of the IL-23 subunit p19 induces multiorgan inflammation, runting, infertility, and premature death.. J Immunol.

[13] Chan JR, Blumenschein W, Murphy E, Diveu C, Wiekowski M (2006). IL-23 stimulates epidermal hyperplasia via TNF and IL-20R2-dependent mechanisms with implications for psoriasis pathogenesis.. J Exp Med.

[14] Zheng Y, Danilenko DM, Valdez P, Kasman I, Eastham-Anderson J (2007). Interleukin-22, a T(H)17 cytokine, mediates IL-23-induced dermal inflammation and acanthosis.. Nature.

[15] Lee E, Trepicchio WL, Oestreicher JL, Pittman D, Wang F (2004). Increased expression of interleukin 23 p19 and p40 in lesional skin of patients with psoriasis vulgaris.. J Exp Med.

[16] Piskin G, Sylva-Steenland RM, Bos JD, Teunissen MB (2006). In vitro and in situ expression of IL-23 by keratinocytes in healthy skin and psoriasis lesions: Enhanced expression in psoriatic skin.. J Immunol.

[17] Capon F, di Meglio P, Szaub J, Prescott NJ, Dunster C (2007). Sequence variants in the genes for the interleukin-23 receptor (IL23R) and its ligand (IL12B) confer protection against psoriasis.. Hum Genet.

[18] Cargill M, Schrodi SJ, Chang M, Garcia VE, Brandon R (2007). A large-scale genetic association study confirms IL12B and leads to the identification of IL23R as psoriasis-risk genes.. Am J Hum Genet.

[19] Gottlieb AB, Cooper KD, McCormick TS, Toichi E, Everitt DE (2007). A phase 1, double-blind, placebo-controlled study evaluating single subcutaneous administrations of a human interieukin-12/23 monoclonal antibody in subjects with plaque psoriasis.. Cur Med Res Opin.

[20] Kauffman CL, Aria N, Toichi E, McCormick TS, Cooper KD (2004). A phase I study evaluating the safety, pharmacokinetics, and clinical response of a human IL-12 p40 antibody in subjects with plaque psoriasis.. J Invest Dermatol.

[21] Krueger GG, Langley RG, Leonardi C, Yeilding N, Guzzo C (2007). A human interleukin-12/23 monoclonal antibody for the treatment of psoriasis.. N Engl J Med.

[22] Toichi E, Torres G, McCormick TS, Chang T, Mascelli MA (2006). An anti-IL-12p40 antibody down-regulates type 1 cytokines, chemokines, and IL-12/IL-23 in psoriasis.. J Immunol.

[23] Teunissen MB, Koomen CW, Malefyt RD, Wierenga EA, Bos JD (1998). Interleukin-17 and interferon-gamma synergize in the enhancement of proinflammatory cytokine production by human keratinocytes.. J Invest Dermatol.

[24] Wilson NJ, Boniface K, Chan JR, McKenzie BS, Blumenschein WM (2007). Development, cytokine profile and function of human interleukin 17-producing helper T cells.. Nature Immunol.

[25] Nograles KE, Zaba LC, Guttman-Yassky E, Fuentes-Duculan J, Suarez-Farinas M (2008). Th17 cytokines interleukin (IL)-17 and IL-22 modulate distinct inflammatory and keratinocyte-response pathways.. Br J Dermatol.

[26] Zaba LC, Cardinale I, Gilleaudeau P, Sullivan-Whalen M, Suarez-Farinas M (2007). Amelioration of epidermal hyperplasia by TNF inhibition is associated with reduced Th17 responses.. J Exp Med.

[27] Kolls JK, Linden A (2004). Interleukin-17 family members and inflammation.. Immunity.

[28] Ma HL, Liang S, Li J, Napierata L, Brown T (2008). IL-22 is required for Th17 cell-mediated pathology in a mouse model of psoriasis-like skin inflammation.. J of Clin Invest.

[29] Boniface K, Guignouard E, Pedretti N, Garcia M, Delwail A (2007). A role for T cell-derived interleukin 22 in psoriatic skin inflammation.. Clin Exp Immunol.

[30] Sa M, Valdez PA, Wu JF, Jung K, Zhong F (2007). The effects of IL-20 subfamily cytokines on reconstituted human epidermis suggest potential roles in cutaneous innate defense and pathogenic adaptive immunity in psoriasis.. J Immunol.

[31] Wolk K, Witt E, Wallace E, Docke WD, Kunz S (2006). IL-22 regulates the expression of genes responsible for antimicrobial defense, cellular differentiation, and mobility in keratinocytes: a potential role in psoriasis.. Eur J Immunol.

[32] Nograles KE, Zaba LC, Shemer A, Fuentes-Duculan J, Cardinale I (2009). IL-22-producing “T22” T cells account for upregulated IL-22 in atopic dermatitis despite reduced IL-17-producing T(H)17 T cells.. J Allergy Clin Immunol.

[33] Bos JD, Hulsebosch HJ, Krieg SR, Bakker PM, Cormane RH (1983). Immunocompetent Cells in Psoriasis - Insitu Immunophenotyping by Monoclonal-Antibodies.. Arch Dermatol Res.

[34] Vissers WH, Arndtz CH, Muys L, Van Erp PE, De Jong EM (2004). Memory effector (CD45RO+) and cytotoxic (CD8+) T cells appear early in the margin zone of spreading psoriatic lesions in contrast to cells expressing natural killer receptors, which appear late.. Br J Dermatol.

[35] Fan X, Yang S, Huang W, Wang ZM, Sun LD (2008). Fine mapping of the psoriasis susceptibility locus PSORS1 supports HLA-C as the susceptibility gene in the Han Chinese population.. Plos Genet.

[36] Nair RP, Stuart PE, Nistor I, Hiremagalore R, Chia NVC (2006). Sequence and haplotype analysis supports HLA-C as the psoriasis susceptibility 1 gene.. Am J Hum Genet.

[37] van der Loos CM (2008). Multiple immunoenzyme staining: Methods and visualizations for the observation with spectral imaging.. J Histochem & Cytochem.

[38] Streeck H, Cohen KW, Jolin JS, Brockman MA, Meier A (2008). Rapid ex vivo isolation and long-term culture of human Th17 cells.. J Immunol Methods.

[39] Duhen T, Geiger R, Jarrossay D, Lanzavecchia A, Sallusto F (2009). Production of interleukin 22 but not interleukin 17 by a subset of human skin-homing memory T cells.. Nature Immunol.

[40] Trifari S, Kaplan CD, Tran EH, Crellin NK, Spits H (2009). Identification of a human helper T cell population that has abundant production of interleukin 22 and is distinct from T-H-17, T(H)1 and T(H)2 cells.. Nature Immunol.

[41] Bos JD, Hagenaars C, Das PK, Krieg SR, Voorn WJ (1989). Predominance of Memory T-Cells (CD4+, CDw29+) Over Naive T-Cells (CD4+, CD45R+) in Both Normal and Diseased Human-Skin.. Arch Dermatol Res.

[42] Johnston A, Gudjonsson JE, Sigmundsdottir H, Love TJ, Valdimarsson H (2004). Peripheral blood T cell responses to keratin peptides that share sequences with streptococcal M proteins are largely restricted to skin-homing CD8(+) T cells.. Clin Exp Immunol.

[43] Lowes MA, Kikuchi T, Fuentes-Duculan J, Cardinale I, Zaba LC (2008). Psoriasis vulgaris lesions contain discrete populations of Th1 and Th17 T cells.. J. Invest Dermatol.

[44] Kryczek I, Bruce AT, Gudjonsson JE, Johnston A, Aphale A (2008). Induction of IL-17(+) T cell trafficking and development by IFN-gamma: Mechanism and pathological relevance in psoriasis.. J Immunol.

[45] Hueber AJ (2010). Cutting Edge: Mast Cells Express IL-17A in Rheumatoid Arthritis Synovium.. J Immunol.

[46] de Boer OJ, van der Meer JJ, Teeling P, van der Loos CM, Idu MM (2010). Differential expression of interleukin-17 family cytokines in intact and complicated human atherosclerotic plaques.. J Pathol.

[47] Ikeda K, Nakajima H, Suzuki K, Kagami SI, Hirose K (2003). Mast cells produce interleukin-25 upon Fc epsilon RI-mediated activation.. Blood.

[48] Weaver CT, Hatton RD, Mangan PR, Harrington LE (2007). IL-17 family cytokines and the expanding diversity of effector T cell lineages.. Annu Rev Immunol.

[49] Liang SC, Tan XY, Luxenberg DP, Karim R, Dunussi-Joannopoulos K, Collins M (2006). Interleukin (IL)-22 and IL-17 are coexpressed by Th17 cells and cooperatively enhance expression of antimicrobial peptides.. J Exp Med.

[50] Yang XX, Chang SH, Park H, Nurieva R, Shah B, Acero L (2008). Regulation of inflammatory responses by IL-17F.. J Exp Med.

[51] Zaba LC, Fuentes-Duculan J, Eungdamrong NJ, Abello MV, Novitskaya I (2009). Psoriasis Is Characterized by Accumulation of Immunostimulatory and Th1/Th17 Cell-Polarizing Myeloid Dendritic Cells.. J Invest Dermatol.

[52] Fuschiotti P, Medsger TA, Morel PA (2009). Effector CD8+ T Cells in Systemic Sclerosis Patients Produce Abnormally High Levels of Interleukin-13 Associated With Increased Skin Fibrosis.. Arthritis Rheum.

[53] Friese MA, Fugger L (2009). Pathogenic CD8(+) T Cells in Multiple Sclerosis.. Ann Neurol.

